# Multi-charge transfer from photodoped ITO nanocrystals†

**DOI:** 10.1039/d1na00656h

**Published:** 2021-09-30

**Authors:** Michele Ghini, Andrea Rubino, Andrea Camellini, Ilka Kriegel

**Affiliations:** Department of Nanochemistry, Istituto Italiano di Tecnologia Via Morego 30 16163 Genova Italy; Dipartimento di Chimica e Chimica Industriale, Università degli Studi di Genova Via Dodecaneso 31 16146 Genova Italy; Functional Nanosystems, Istituto Italiano di Tecnologia (IIT) Via Morego 30 16163 Genova Italy Ilka.Kriegel@iit.it

## Abstract

Metal oxide nanocrystals are emerging as an extremely versatile material for addressing many of the current challenging demands of energy-conversion technology. Being able to exploit their full potential is not only an advantage but also a scientific and economic ambition for a more sustainable energy development. In this direction, the photodoping of metal oxide nanocrystals is a very notable process that allows accumulating multiple charge carriers per nanocrystal after light absorption. The reactivity of the photodoped electrons is currently the subject of an intense study. In this context, the possibility to extract efficiently the stored electrons could be beneficial for numerous processes, from photoconversion and sunlight energy storage to photocatalysis and photoelectrochemistry. In this work we provide, *via* oxidative titration and optical spectroscopy, evidence for multi-electron transfer processes from photodoped Sn : In_2_O_3_ nanocrystals to a widely employed organic electron acceptor (F4TCNQ). The results of this study disclose the potential of photodoped electrons to drive chemical reactions involving more than one electron.

## Abstract 2

Photodoped ITO nanocrystals (NCs) enables multi-electron transfer processes unveiled by F4TCNQ titration analysis. Metal oxide NCs are emerging as extremely versatile materials for energy storage and conversion technologies.

## Graphical abstract

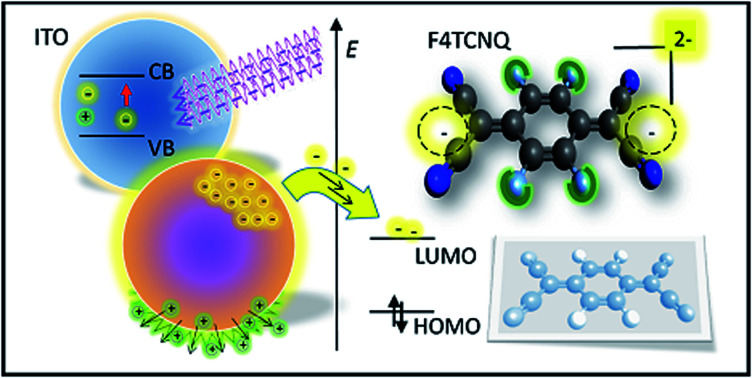

## Introduction

Metal oxide (MO) semiconductors are inorganic materials of great interest in the field of optoelectronic applications for energy-related technology.^1^ They offer an ideal combination of several properties ranging from environmental stability, chemical tunability^2^ to optical transparency and good charge mobility.^3^ The ability to modulate their charge carrier density through aliovalent substitutional doping and post-synthesis methods boosts the implementation of doped MO nanocrystals (NCs), such as Sn-doped In_2_O_3_ (Indium Tin Oxide [ITO]) NCs.^4–7^ Doped MO NCs, in fact, combine both the ability to absorb NIR radiation through the excitation of doping- and size-dependent localized surface plasmon resonances (LSPR) and the potential to store multiple delocalized photo-excited carriers.^8–10^

Photodoping, a light-driven charge accumulation of electrons induced by multiple absorption events of high-energy photons (beyond the bandgap of MO semiconductors), emerged as a contactless and promising tool to promote the photo-conversion process.^7,11–16^ The photodoping of ITO NCs in the presence of a sacrificial hole scavenger, such as ethanol, under anaerobic conditions, results in the reversible accumulation of tens to hundreds of electrons per single NC.^16^ The reversibility of the photodoping process (and therefore the removal of photo-chemically excited electrons), already well demonstrated, was also analysed *via* potentiometric titration with the addition of a molecular oxidant to the colloidal suspension of MO NCs.^11,12,14,16,17^ Due to the proportionality between the plasmonic peak energy *ω*_LSPR_ and the free carrier density,^18^ the storage and depletion of photodoped electrons upon photodoping and oxidative titration can also be performed by monitoring the spectral evolution of the LSPR.^7,14–16,19–21^ In this context, the opportunity to access multiple charge transfer events of photodoped electrons would be enormously relevant in terms of efficiency, not only for photovoltaic energy conversion purposes, but also in applications such as batteries, or implementations in photoelectrochemistry and photocatalysis.^22–24^ The transfer of more than one electron per unit of active component (*i.e.* MO NCs) implies an increase in energy density, which also translates into a reduced amount of materials to be employed and lower costs. This energetic and economic principle, perfectly in line also with current sustainability policies, is obviously valid for the redox transformations of electrochemical cells and for catalytic reactions.^25^ Regardless of whether the transfer takes place in solution or in solid systems such as electrodes, the participation of multiple charges can enhance the performance in terms of reaction yield and kinetics. Such a beneficial process has already been observed in many reports, as in the case of gold nanoparticles for CO_2_ conversion or organic donor–acceptor systems capable of proton-coupled multi-electron transfer.^24,26,27^ Similarly, colloidal semiconductor nanocrystals have been demonstrated as being able to release multiple electrons, after the absorption of light, which can then interact with organic compounds or be suitably stored for the subsequent production of current.^28^ Until now, only a few MO related studies have specifically addressed the possibility of multiple electron transfer of photo-excited electrons. For instance, ZnO and TiO_2_ NCs have found successful use in electrochemical applications such as the oxygen reduction reaction, which typically involve multiple electron transfer processes.^13,29–31^

In this work, *via* oxidative titration and optical spectroscopy, we investigate the ability of ITO NCs to provide multiple transfers of electrons accumulated after the photodoping process. Specifically, to address multi-electron oxidation we made use of a chemical compound capable of undergoing double ionization, namely 2,3,5,6-tetrafluoro-7,7,8,8-tetracyanoquinodimethane (F4TCNQ). The concentration dependent evolution of the F4TCNQ ionized species, with their peculiar spectroscopic features, will help in discerning the mechanism of the charge transfer as a single or a multi-electron phenomenon.

## Experimental section

For the synthesis of nanocrystals, we adopted a continuous growth mechanism starting from a solution of precursors, indium(iii) acetate (Sigma-Aldrich) and tin(iv) acetate (Sigma-Aldrich) in a 1 : 9 = Sn : In ratio. The two acetates were left in oleic acid (Sigma Aldrich) at 150 °C under N_2_ for several hours. The precursors were then slowly injected (at a rate of 0.30 mL min^−1^) with a syringe pump into 13 mL of oleyl alcohol at 290 °C. The solution was then washed (twice) with ethanol (∼12 mL) and centrifuged at 7300 rpm for 10 min. Finally, the colloidal nanoparticles were dispersed in hexane (Sigma Aldrich) as a stock solution with a concentration of 13 mg mL^−1^. F4TCNQ titrants were prepared by dissolving 0.34 mg in 40 mL of anhydrous toluene (Sigma Aldrich). In order to avoid any contact with external oxygen, titrant addition steps were carried out in the inert environment of an argon filled glovebox. For both photodoping and titration experiments, rectangular anaerobic cuvettes with a sealed screw cap, an optical path of 5 mm and a volume of 1.4 mL were used (Starna Scientific). The photodoping process was carried out by illuminating the cuvette containing the solution of ITO NCs dissolved in anhydrous toluene with a UV LED (central wavelength: 300 nm and bandwidth: 20 nm) placed at a distance of 12 mm from the cuvette window (Thorlabs M300L4). The UV power density at the front window of the cuvette was 36.8 mW cm^−2^. The monitoring of the optical response of both the as-prepared ITO NCs upon photodoping and the ITO/F4TCNQ mixture after each titration step was carried out by recording the absorbance spectrum using a UV-Vis-NIR spectrophotometer (Agilent Cary 5000). The quantitative and structural characterization of nanoparticles was carried out *via* inductively coupled plasma mass spectrometry (ICP-OES) and transmission electron microscopy (JEOL JEM-1400Plus – 120 kV TEM/STEM).

## Results and discussion

### (A) ITO NC photodoping

**Fig. 1:**
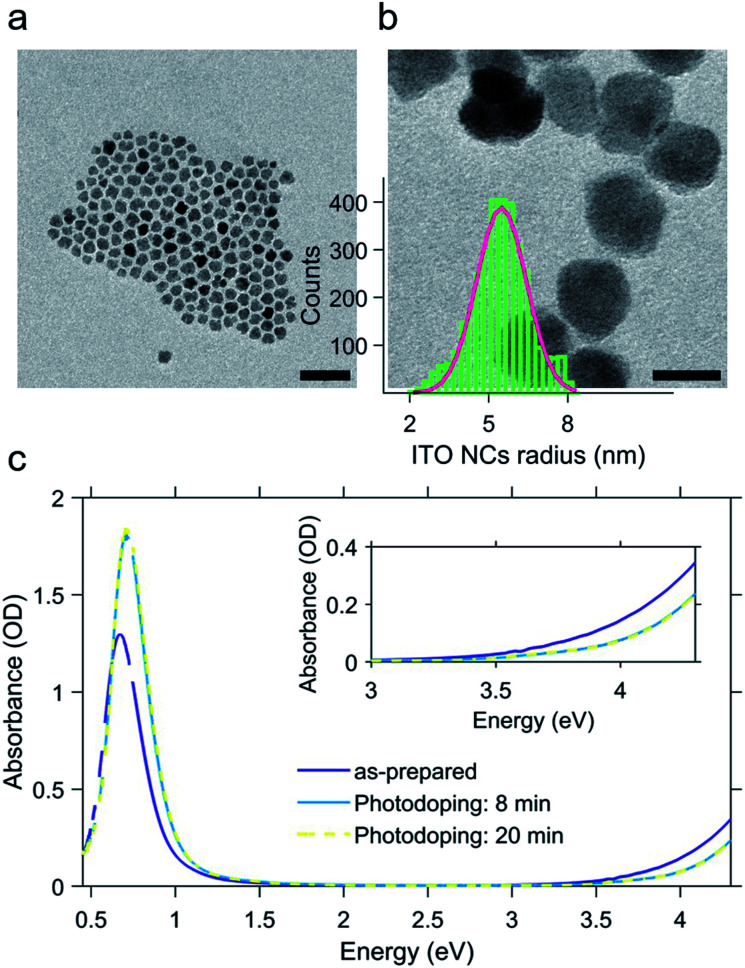
Transmission electron microscopy images of the as-prepared Sn-doped In_2_O_3_ (ITO) nanocrystals; scale bar: 50 nm (a) and 10 nm (b). Panel (b) also shows the nanocrystals' radius size distribution obtained *via* statistical analysis of the transmission electron microscopy images. Absorbance spectra of the as-prepared and photodoped ITO nanocrystals (c) dissolved in anhydrous toluene. Photodoping is performed by illuminated the ITO nanocrystal solution with a UV LED (central wavelength: 300 nm) with increasing exposure time with respect to the as-prepared conditions (8 and 20 minutes). Inset of panel c highlights the effect of photodoping on the band edge absorption (Burstein–Moss effect).

The ITO NCs analyzed in this work have a diameter of about 11 nm (Fig. 1a and b) and are colloidal particles stabilized by organic ligands. More precisely, the organic part consists of oleate molecules. In view of the study on electro-optic behavior through titration, we took an aliquot of the colloidal solution in hexane and let the nanoparticles dry and we transferred them into a glovebox, where we prepared a solution in toluene anhydrous (∼0.1 × 10^−9^ mol L^−1^). The solvent exchange is dictated by the low solubility of F4TCNQ molecules, employed as a diagnostic tool for assessing the electron transfer, and inert atmosphere conditions are strictly needed for the photodoping process occurring in a liquid environment.

In Fig. 1c, we report the results of the photodoping experiment on ITO nanocrystals. As can be seen from the absorption spectra recorded upon a gradual increase of illumination times, the UV excitation alters the optical response of ITO NCs. In particular, spectral changes affect both the plasmonic peak and the bandgap. As the exposure time is prolonged, the LSPR feature associated with the plasmonic absorption increases in intensity and moves towards higher energies. Similarly, the bandgap onset also undergoes a blue shift. The effect on these absorption properties reaches saturation after 8 minutes of UV exposure. At this point, we want to underline a substantial difference with respect to the usual conditions adopted in the doping treatment with ultraviolet light. In order to both keep the overall charge neutrality and stabilize the accumulated electron density of each nanocrystal, the intervention of a hole quencher (*e.g.* ethanol and methanol) is necessary. In our case, however, the increase in the density of charges and the consequent increase in the plasmonic signal are quite evident without the addition of further compounds to the solution in toluene. Such a behavior indicates that the recombination of the photogenerated charges is most likely suppressed thanks to the presence of the ligands and/or surface states, without the addition of alcohols, as already demonstrated in other cases in the literature^12,13,32–35^ albeit the improbable trace of some residual EtOH molecules absorbed after the NC washing cycles. The bleaching of the band edge absorption is the result of the widening of the bandgap (Burstein–Moss effect^36^). This effect, also reported for different MO NCs (*e.g.* doped indium oxide), further confirms the increase of electron density upon photodoping.

### (B) ITO/F4TCNQ titration

Once the maximum intensity of the LSPR peak reached saturation, we started the quantitative analysis of the number of electrons released by the photodoped ITO NCs by means of successive addition of oxidative titrants. As already mentioned, in this study, we selected F4TCNQ, a well-known electron acceptor with a high value of electron affinity (5.2 eV), widely employed both for fundamental studies of charge transfer processes (*e.g.* photoluminescence quenching) and as a p-dopant of organic materials in hole-transporting layers for photovoltaic applications.^37–39^ More specifically, the characteristic that makes this compound particularly interesting in the analysis of multiple electron transfer is its ability to acquire, in its neutral form, up to two electrons. F4TCNQ is thus able to form two distinct ionized species^40^ with the donor–host environment having lower values of ionization energy (≲5 eV). Moreover, the formation of stable anion and dianion species leads to different spectral features in the optical absorption of the donor–F4TCNQ mixture therefore allowing the possibility to distinguish between single and double ionization processes with routinely available optical measurements. The formation of F4TCNQ dianions is observed in several polymer-dopant systems.^41–43^ Recently, the doping of bithiophene–thienothiophene-based copolymers with F4TCNQ showed an almost complete double ionization of dopant molecules providing an ionization efficiency of 200%, which also resulted in an enhancement of charge carrier transport.^41,44^ To the best of our knowledge double reduction of F4TCNQ has never been reported neither for inorganic-dopant systems nor for photodoped MO NCs-dopant mixtures.

**Fig. 2:**
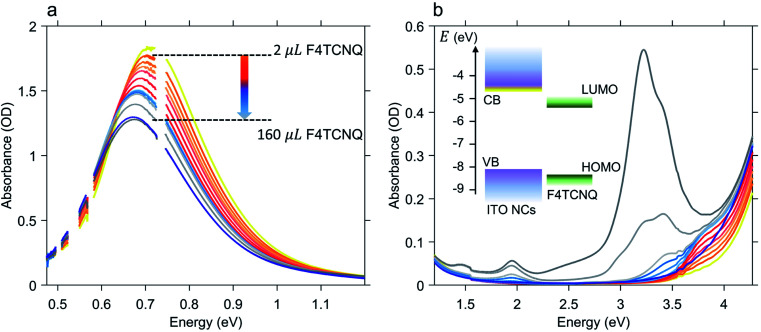
Results of the oxidative titration of photodoped Sn-doped In_2_O_3_ (ITO) nanocrystals. Panel (a) shows the recovery of the localized surface plasmon feature to the as-prepared conditions upon adding increasing amount of F4TCNQ molecules. Panel (b) shows the titration effects induced on the ITO bandgap absorption region and energy levels of ITO NCs and F4TCNQ (VB: valence band, CB: conduction band, HOMO: highest occupied molecular orbital, and LUMO: lowest unoccupied molecular orbital). Dark blue and yellow lines in panels (a) and (b) correspond to the as-prepared and photodoped (exposure time: 20 minutes) ITO NC absorption spectra. During titration, absorption spectra were recorded after adding a total sum of 2, 4, 6, 8, 11, 14, 17, 20, 23, 26, 60 and 160 μL of F4TCNQ to the photodoped ITO NC solution.

Fig. 2 shows the results of the titration experiment on ITO NCs. In order to accurately establish the number of electrons extracted from the ITO NCs, we conducted titration by gradually adding an increasing number of F4TCNQ molecules from a 0.3 mM stock solution and monitoring over time, through absorption, the formation of reduced F4TCNQ species (molar ratio between 88 and 98 mol%, see Table S1 in the ESI† file). Fig. 2a displays the effects of the addition of the electron acceptor on the LSPR peak of the ITO NCs in the NIR range. In this case, we observe that through titration the system re-establishes its as-prepared conditions: the LSPR peak decreases in intensity and undergoes a red shift with increasing amount of F4TCNQ. Both are signatures of the reduction of charge density in ITO NCs, which have reacted with the F4TCNQ molecules. The photogenerated and charged-compensated electrons in the post-synthetic photodoping treatment are more reactive with respect to the electrons present due to the aliovalent doping after the incorporation of tin elements in the indium oxide, even under mild oxidation conditions.^16,45^ Taking into consideration, instead, the spectral window in the UV-Vis range, as shown in Fig. 2b, we can see a second effect on the absorption of the ITO. In conjunction with the recovery of the LSPR signal, the absorption band edge also tends to return to the pre-photodoping spectral shape by compensating for the blue shift originating from the Burstein–Moss effect. Interestingly, in this region, the appearance of a second contribution at around 3.7 eV is evident. This contribution to the overall absorption recovery increases in its intensity only during the first titrant additions (orange and red spectra in Fig. 2b) and, as detailed below, represents the formation of an F4TCNQ dianion in the ITO NCs/F4TCNQ mixture. Moreover, in the latest addition of titrants (>100 μL), dilution effects start to play a role in the decrease of the LSPR peak intensity. At this stage, it is therefore convenient to switch the focus towards the information obtained from the analysis of the neutral and ionized species, following systematically the titration process.

### (C) Multi-electron transfer

As previously discussed, the titration experiment allows for the quantification of the number of charges that can be extracted per photodoped nanocrystal and the analysis of the number of electrons involved in the transfer process. For this purpose, we want to recall briefly the established spectral features associated with F4TCNQ species and their photo-physics. The absorption peak of the neutral molecule has a maximum at around 3.2 eV, whereas the peak of its anion shows a maximum at around 3 eV and two characteristic peaks between 1.3 and 2 eV. The double ionized molecule (dianion) presents an absorption peak at around 3.7–3.8 eV. In addition, this compound is able to react through integer charge transfer and/or form charge transfer complexes.^46,47^ The ionized species of F4TCNQ are also particularly reactive and can interact with each other or even dimerize.^48,49^ All these transformations can generate new optically active transitions that “intervene” in the absorption with new peaks that may appear along with those already mentioned.

**Fig. 3:**
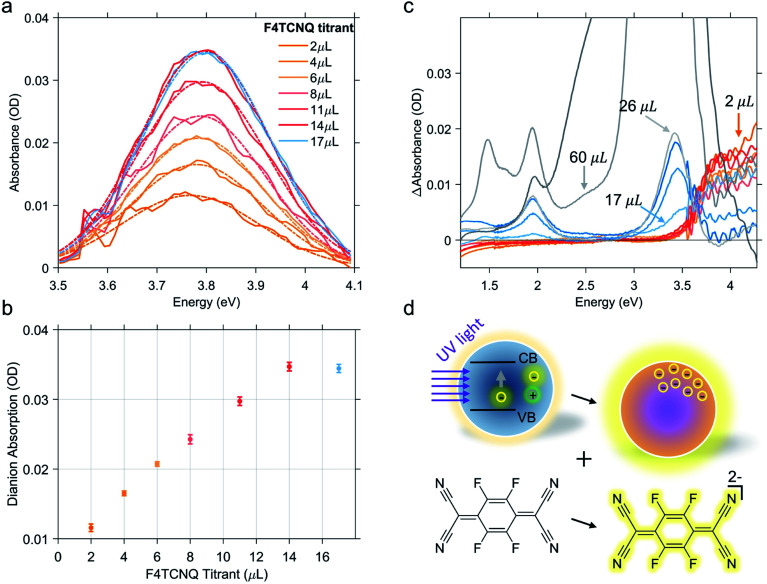
F4TCNQ dianion contribution to the absorption spectra of the ITO NCs/F4TCNQ mixture. Dashed-dotted line is obtained by fitting the dianion contribution with a Gaussian function (a). Dianion contribution increases linearly until the addition of 14 μL of F4TCNQ (b). Panel (c) represents the incremental effect of titration on the absorption spectra of the ITO NCs/F4TCNQ mixture. Differential absorbance ΔAbsorbance is obtained by subtracting the spectrum from the previous titration step from each curve. (d) Pictorial representation of the two electron transfer process from photodoped ITO NCs to F4TCNQ molecules.

Hence, in the titration process, the absorption spectra recorded from the solution of photodoped ITO NCs/F4TCNQ molecules contains the contributions from all the species and charge transfer states that can be formed after the reduction with electrons accumulated in ITO NCs. The spectra shown in Fig. 2b are, in fact, a convolution of several contributions. As already observed in Fig. 2, the superposition of the signals coming from the oxidant and the reductant concerns the UV-Vis region. Clearly, the absorption measurements recorded right after the addition of F4TCNQ to the solution of photodoped ITO NCs revealed the presence of the dianion alone among the F4TCNQ species (see Fig. S1 in the ESI† file). In order to correctly analyze the behavior of the titrant and separate it from the response of the ITO NCs, we fitted the experimental absorption spectra in the UV range with a polynomial function, which takes into account the ITO bandgap recovery in the UV range upon titration (see Fig. S2 in the ESI†). Fig. 3a displays the resulting peak obtained by subtracting this latter effect. The characteristic F4TCNQ dianion peak appears and increases in intensity with subsequent additions up to 14 μL of titrant solution (Fig. 3b). This result demonstrates how photodoped ITO NCs are capable of a two-electron transfer that can, directly, double ionize F4TCNQ. This is the first evidence of such an optoelectronic response from ITO NCs.

Continuing with the titrant additions, the double reduction effect reaches a saturation level. Fig. 3c shows the sequence of spectra of the titration experiments. In this case, the spectrum from the previous titration step was subtracted from each curve. In this way, we wanted to highlight the change in the F4TCNQ ionization regime. In fact, after the addition of 14 μL, the formation of the neutral titrant species becomes visible. This suggests the interruption not only of the two-electron transfer, but of the ionization in general. Actually, a contribution relative to the anionic species is also observed in the visible region, which increases, albeit slightly. This second observation could also indicate the possibility that the transfer of photogenerated electrons continue with single transfers. As already mentioned, however, F4TCNQ can incur different types of reactions, including back donations and dimerization as suggested by an isosbestic point at 3.6 eV (see Fig. 3c) between the region of neutral and dianion absorption. For this reason, we refrain from interpreting this last stage of the titration in terms of the number of electrons extracted. In this step, moreover, even minimal dilution effects can come into play and affect the overall optical response of the mixture.

As a counter proof of the behavior analyzed so far actually deriving from the interactions between the electrons accumulated through photodoping and the electron acceptor molecules, we also carried out a control experiment on a similar ITO NC sample, adding the titrant molecules before the photodoping. The result, shown in Fig. S3 of the ESI,† indicates that adding F4TCNQ to the solution containing ITO NCs does not cause any ionization and we can observe just the neutral-species related peak increasing in intensity according to the added volume of F4TCNQ solution. Electrons added through tin doping are more stable and do not participate in the titration. Surprisingly, illuminating, with a LED, the mixture containing F4TCNQ and ITO NCs we directly observed the formation of the dianionic species without any relevant changes in the ITO plasmonic peak. This behavior supposes a substantial difference between the kinetics of photodoping and the reactivity of the photodoped electrons, in favor of the latter. However, in this case, we cannot properly analyze the result considering the fact that prolonged illumination with ultraviolet light not only contributes to photodoping but can also initiate a degradation process of the organic titrant. In any case, what is also evident is that as the neutral peak disappears, either because of the dianion formation or because of the photodegradation, the ITO NC photodoping signal starts to appear. The plasmonic peak increases since no more neutral titrant molecules are available for the oxidation.

Finally, considering the starting concentration of the ITO nanoparticles, the concentration of the F4TCNQ molecules added until the appearance of the neutral peak and the transfer of two electrons per F4TCNQ molecule, we were able to estimate the number of electrons extracted per ITO NC (more details on the calculation can be found in the ESI†). The result obtained reveals a transfer of ∼123 electrons per nanocrystal. Interestingly the number of electrons is in fair accordance with the order of magnitude that can be extrapolated from the relative variation of the plasmon peak energy, after photodoping, as a function of the F4TCNQ equivalents (see Fig. S4a†). This agreement is also evident from the analysis of the linear dependence of *ω*_LSPR_^2^ on the number of electrons extracted as reported in Fig. S4b.† Given the complexity of the final titration phase, it is also possible that this number could be an underestimate of the real number. In any case, the value obtained is an indication of the enormous potential of photodoped ITO NCs, as a multi-electron extraction platform. This direct evidence certainly deserves further study, taking into account several factors: first of all, the optimization of the system in terms of materials (size, shape and level of aliovalent doping), operating conditions of the photodoping procedure and finally the stabilization of the charges with suitable hole scavengers.^29^ The potential to enhance energy density through multi-charge transfer processes makes these systems extremely competitive in the current research market for energy materials.

## Conclusions

In summary, in this work we investigated the possibility of using photodoped electrons of ITO NCs for multiple charge transfer processes *via* titration with F4TCNQ molecules. Being, the latter, a compound capable of single and/or double ionization, we were able to assess the number of electrons associated with the reduction process under our operating conditions. The result we report revealed several strengths of the nanocrystal-based system. The synthesized nanoparticles are able to store new electrons after ultraviolet illumination without the need for additional hole-scavengers. The photodoped electrons can then ionize the F4TCNQ molecules with a transfer of two electrons thus showing the potential of these highly reactive electrons to be employed as efficient multi-electron photocatalysts. The photodoped ITO NCs can release more than 100 electrons per NC, demonstrating strong competitiveness, for example in the new generation of high-density batteries. The photodoping process provides a non-invasive method and the nanocrystals retain their stability, making them attractive candidates for the efficient development of light-driven energy conversion/storage applications and for multi-electron photocatalysis.

## Author contributions

M. G.: conceptualization, investigation, formal analysis, and writing – review & editing. A. R.: conceptualization and writing – original draft – review & editing. A. C.: conceptualization, formal analysis, and writing – review & editing. I. K.: supervision, conceptualization, and writing – review & editing.

## Conflicts of interest

There are no conflicts to declare.

## Supplementary Material

NA-003-D1NA00656H-s001

## References

[cit1] Maduraiveeran G., Sasidharan M., Jin W. (2019). Prog. Mater. Sci..

[cit2] Gatti T., Lamberti F., Mazzaro R., Kriegel I., Schlettwein D., Enrichi F., Lago N., Maria E. D., Meneghesso G., Vomiero A., Gross S. (2021). Adv. Energy Mater..

[cit3] Klein A. (2013). J. Am. Ceram. Soc..

[cit4] Kriegel I., Scotognella F., Manna L. (2017). Phys. Rep..

[cit5] Tandon B., Ghosh S., Milliron D. J. (2019). Chem. Mater..

[cit6] Matsui H., Furuta S., Tabata H. (2014). Appl. Phys. Lett..

[cit7] Ghini M., Curreli N., Camellini A., Wang M., Asaithambi A., Kriegel I. (2021). Nanoscale.

[cit8] Tandon B., Ashok A., Nag A. (2015). Pramana.

[cit9] Ghini M., Yanev E. S., Kastl C., Zhang K., Jansons A. W., Crockett B. M., Koskela K. M., Barnard E. S., Penzo E., Hutchison J. E., Robinson J. A., Manna L., Borys N. J., Schuck P. J., Kriegel I. (2021). Adv. Photonics Res..

[cit10] Kriegel I., Ghini M., Bellani S., Zhang K., Jansons A. W., Crockett B. M., Koskela K. M., Barnard E. S., Penzo E., Hutchison J. E., Robinson J. A., Manna L., Borys N. J., Schuck P. J. (2020). J. Phys. Chem. C.

[cit11] Haase M., Weller H., Henglein A. (1988). J. Phys. Chem..

[cit12] van Dijken A., Meulenkamp E. A., Vanmaekelbergh D., Meijerink A. (2000). J. Phys. Chem. B.

[cit13] Schrauben J. N., Hayoun R., Valdez C. N., Braten M., Fridley L., Mayer J. M. (2012). Science.

[cit14] Liu W. K., Whitaker K. M., Kittilstved K. R., Gamelin D. R. (2006). J. Am. Chem. Soc..

[cit15] Schimpf A. M., Thakkar N., Gunthardt C. E., Masiello D. J., Gamelin D. R. (2014). ACS Nano.

[cit16] Schimpf A. M., Lounis S. D., Runnerstrom E. L., Milliron D. J., Gamelin D. R. (2015). J. Am. Chem. Soc..

[cit17] Brozek C. K., Hartstein K. H., Gamelin D. R. (2016). J. Am. Chem. Soc..

[cit18] Staller C. M., Gibbs S. L., Saez Cabezas C. A., Milliron D. J. (2019). Nano Lett..

[cit19] Gibbs S. L., Staller C. M., Milliron D. J. (2019). Acc. Chem. Res..

[cit20] Faucheaux J. A., Jain P. K. (2013). J. Phys. Chem. Lett..

[cit21] Zhang H., Kulkarni V., Prodan E., Nordlander P., Govorov A. O. (2014). J. Phys. Chem. C.

[cit22] Wu F., Yang H., Bai Y., Wu C. (2020). J. Energy Chem..

[cit23] Chen R., Luo R., Huang Y., Wu F., Li L. (2016). Adv. Sci..

[cit24] Pannwitz A., Wenger O. S. (2019). Chem. Commun..

[cit25] Koper M. T. M. (2011). J. Electroanal. Chem..

[cit26] Yu S., Wilson A. J., Heo J., Jain P. K. (2018). Nano Lett..

[cit27] Kim Y., Smith J. G., Jain P. K. (2018). Nat. Chem..

[cit28] Knowles K. E., Malicki M., Parameswaran R., Cass L. C., Weiss E. A. (2013). J. Am. Chem. Soc..

[cit29] Schimpf A. M., Gunthardt C. E., Rinehart J. D., Mayer J. M., Gamelin D. R. (2013). J. Am. Chem. Soc..

[cit30] Mohamed H. H., Mendive C. B., Dillert R., Bahnemann D. W. (2011). J. Phys. Chem. A.

[cit31] Castillo-Lora J., Delley M. F., Laga S. M., Mayer J. M. (2020). J. Phys. Chem. Lett..

[cit32] Wuister S. F., de Mello Donegá C., Meijerink A. (2004). J. Phys. Chem. B.

[cit33] Völker J., Zhou X., Ma X., Flessau S., Lin H., Schmittel M., Mews A. (2010). Angew. Chem., Int. Ed..

[cit34] Morris-Cohen A. J., Frederick M. T., Lilly G. D., McArthur E. A., Weiss E. A. (2010). J. Phys. Chem. Lett..

[cit35] Cohn A. W., Schimpf A. M., Gunthardt C. E., Gamelin D. R. (2013). Nano Lett..

[cit36] Hamberg I., Granqvist C. G., Berggren K.-F., Sernelius B. E., Engström L. (1984). Phys. Rev. B: Condens. Matter Mater. Phys..

[cit37] Yu Z., Zhang Y., Jiang X., Li X., Lai J., Hu M., Elawad M., Gurzadyan G. G., Yang X., Sun L. (2017). RSC Adv..

[cit38] Khan A. A., Azam M., Eric D., Liang G., Yu Z. (2020). J. Mater. Chem. C.

[cit39] Tyagi P., Tuli S., Srivastava R. (2015). J. Chem. Phys..

[cit40] Panja S., Kadhane U., Andersen J. U., Holm A. I. S., Hvelplund P., Kirketerp M.-B. S., Nielsen S. B., Støchkel K., Compton R. N., Forster J. S., Kilså K., Nielsen M. B. (2007). J. Chem. Phys..

[cit41] Kiefer D., Kroon R., Hofmann A. I., Sun H., Liu X., Giovannitti A., Stegerer D., Cano A., Hynynen J., Yu L., Zhang Y., Nai D., Harrelson T. F., Sommer M., Moulé A. J., Kemerink M., Marder S. R., McCulloch I., Fahlman M., Fabiano S., Müller C. (2019). Nat. Mater..

[cit42] Kroon R., Kiefer D., Stegerer D., Yu L., Sommer M., Müller C. (2017). Adv. Mater..

[cit43] Kataeva O., Metlushka K., Ivshin K., Kiiamov A., Alfonsov V., Khrizanforov M., Budnikova Y., Sinyashin O., Krupskaya Y., Kataev V., Büchner B., Knupfer M. (2018). Eur. J. Inorg. Chem..

[cit44] Lüssem B. (2019). Nat. Mater..

[cit45] Goings J. J., Schimpf A. M., May J. W., Johns R. W., Gamelin D. R., Li X. (2014). J. Phys. Chem. C.

[cit46] Theurer C. P., Valencia A. M., Hausch J., Zeiser C., Sivanesan V., Cocchi C., Tegeder P., Broch K. (2021). J. Phys. Chem. C.

[cit47] Stanfield D. A., Wu Y., Tolbert S. H., Schwartz B. J. (2021). Chem. Mater..

[cit48] Faulques E., Leblanc A., Molinié P., Decoster M., Conan F., Sala-Pala J. (1997). J. Phys. Chem. B.

[cit49] Ma L., Hu P., Jiang H., Kloc C., Sun H., Soci C., Voityuk A. A., Michel-Beyerle M. E., Gurzadyan G. G. (2016). Sci. Rep..

