# Supplementary material for: Multi-charge transfer from photodoped ITO nanocrystals
Source: Nanoscale Adv. 2021 Sep 30;3(23):6628–34. doi: 10.1039/d1na00656h (PMC8610084; doi:10.1039/d1na00656h)
Supplement: NA-003-D1NA00656H-s001 [file NA-003-D1NA00656H-s001.pdf]

Electronic Supplementary Material (ESI) for Nanoscale Advances.
This journal is © The Royal Society of Chemistry 2021

SUPPLEMENTARY INFORMATION

Multi-charge Transfer from Photodoped ITO Nanocrystals

Michele Ghini,[a,b] Andrea Rubino,[c] Andrea Camellini,[c] Ilka Kriegel[c]*

[a] *Department of Nanochemistry, Istituto Italiano di Tecnologia, via Morego 30, 16163 Genova, Italy*

[b] *Dipartimento di Chimica e Chimica Industriale, Università degli Studi di Genova, Via Dodecaneso 31, 16146 Genova, Italy*

[c] *Functional Nanosystems, Istituto Italiano di Tecnologia (IIT), via Morego 30, 16163 Genova, Italy*

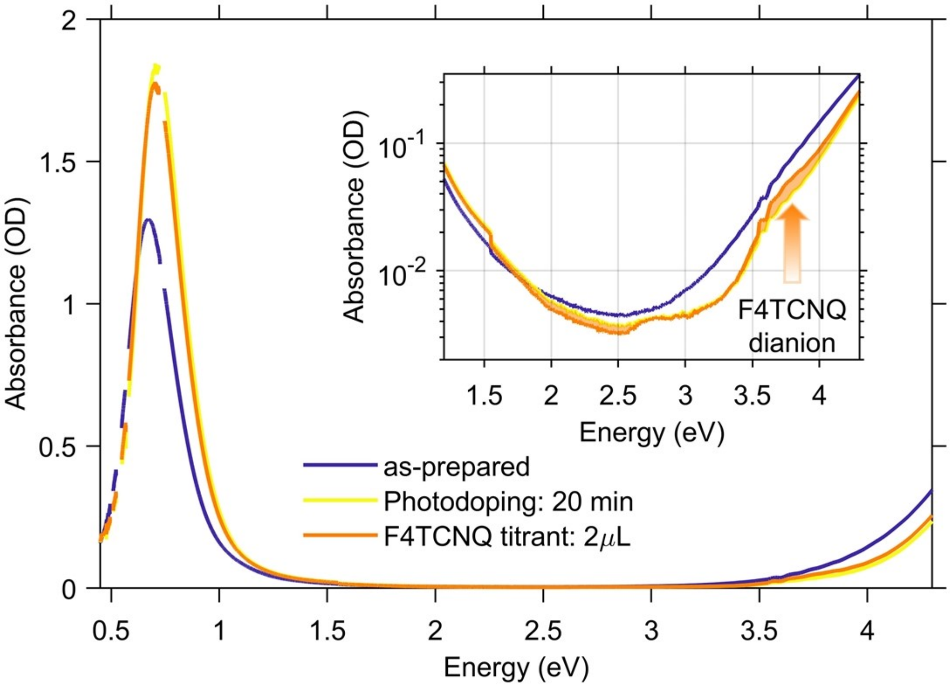

Figure S1 Absorbance spectra of as prepared ITO NCs, photodoped ITO NCs (UV exposure time 20min) and ITO NCs/F4TCNQ mixture after the addition of the first titrant aliquot (2 μL, 88 mol%). Orange shaded area in the inset highlight the effect of the first titration step with respect to the photodoped ITO NCs solution. Only a small contribution between 3.5 and 4eV can be distinguished.

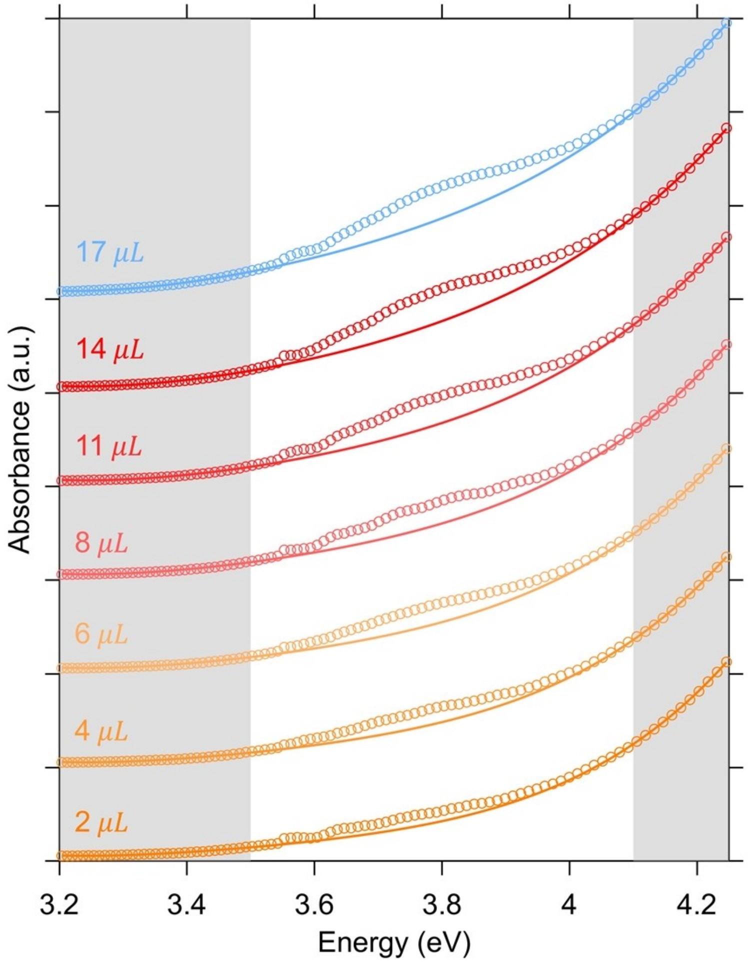

Figure S2 Absorption spectra of photodoped ITO NCs after the addition of increasing amount of F4TCNQ titrant solution (0.3mM in anhydrous Toluene). In order to isolate the absorption of the F4TCNQ dianion from the recovery of the band edge we performed a 4th order fit of the absorption spectra. Fitting was performed by taking into account only those data points outside the region of dianion absorption (grey shaded areas).

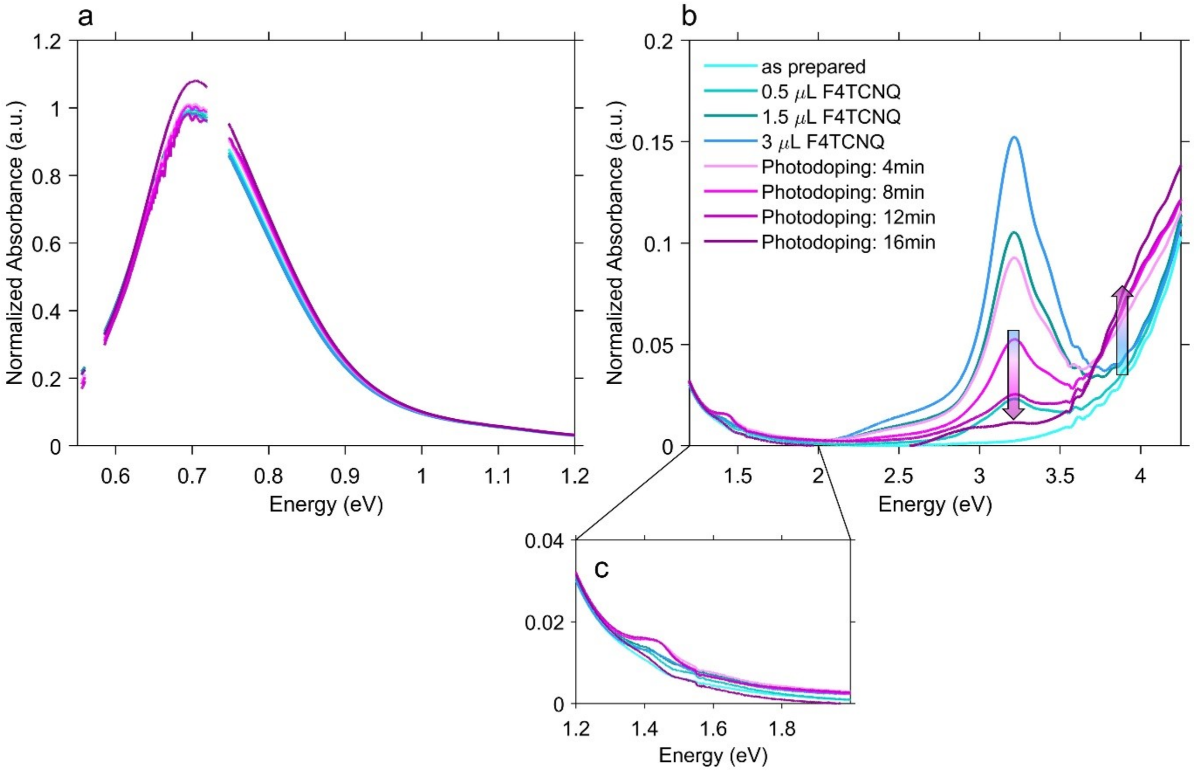

Figure S3 Normalized absorbance spectra of as prepared ITO NCs, ITO NCs/F4TCNQ mixture for increasing amounts of F4TCNQ (0.5, 1.5, 3 μL) before any photodoping process. Absorbance spectra in magenta color scale show the effect of photodoping on ITO NCs/F4TCNQ mixture after the addition of 3μL of F4TCNQ. Panel (a) and panels (b, c) show the evolution of the spectra in the plasmonic and band gap regions, respectively. Arrows in Panel (b) display the effect of photodoping on the ITO NCs/F4TCNQ mixture. Panel (c) show a zoom in the spectral region of F4TCNQ anion absorption.

Number of electrons extracted via Oxidative Titration of photodoped ITO NCs

We describe here in details the procedure followed to estimate the number of electrons extracted from each photodoped ITO NC upon oxidative titration. Given the concentration of titrant solution ($c =$ 0.085 mg/mL) and the molecular mass of F4TCNQ molecules (276.15 g/mol), we can calculate the amount of moles of titrant molecules (n_{F4TCNQ}) added in the cuvette at each step of the experiment, as a function of the injected volume: $n_{F4TCNQ}(V) = cV/276.15$ (see Table S1). On the other hand, we calculated the weight of the average ITO NC from the volume of the unit cell (1.0355 nm^3), the weight of Sn, In and O atoms and the average radius of the NCs from TEM images (see Fig1b). In addition, from ICP-OES mass spectroscopy data we estimated the concentration of NCs present in the solution in order to extrapolate the number of ITO NCs moles (n_{NC}). Then, by normalizing n_{F4TCNQ} with the moles of NCs (n_{NC}) present in the colloidal solution, we obtain the number of oxidizing molecule that reacted with each NC: $n_{reacted}(V) = n_{F4TCNQ}(V)/n_{NC}$. Finally, in order to estimate the number extracted photo-electrons for each NC, we multiply $n_{reacted}$ by a factor two or one, depending on the kind of reaction taking place (identified from dianion or anion peaks, respectively).

Thanks to the spectroscopic analysis showed in **Fig. 3**, we can safely assume that up to $V_1 =$ 14 μL (volume of added F4TCNQ titrants) only dianion reactions occur, since no signatures of neutral nor anion peaks are present. We noticed that the next addition, $V_2 =$ 17 μL, acts as a threshold value in the evolution of the reaction. At V_2, anion and neutral peaks start to appear in the UV-Vis range, and progressively increase with the further additions of titrants. After V_2, even if the LSPR peak continues to decrease and red-shift, suggesting that further extraction of stored electrons is still possible, the non-complete reaction of F4TCNQ molecules makes the analysis too complex to be carried on. Here, we counted two electrons for each F4TCNQ molecule which reacted until the midpoint between V_1 and V_2 ($Vmid = \frac{V1 + V2}{2} =$ 15.5 μL). Moreover, since the appearance of anion peaks indicates that the carrier extraction process still occurs at least up to V_2, we then added one extra electron for each molecule between $Vmid$ and V_2. Therefore the total number of

electron extracted from each ITO NCs can be estimated by: $2e^- \times n_{reacted}(V_{mid}) + 1e^- \times (n_{reacted}(V_2) - n_{reacted}(V_{mid})) \approx$ 123.

	Moles of ITO NCs (n_{NC}) [10⁻⁹ mol]		
	0.08		
**Added Volume of F4TCNQ [μL]**	**Moles of F4TCNQ (n_{F4TCNQ}) [10⁻⁹ mol]**	**mol %**	$n_{reacted} = n_{F4TCNQ}/n_{N}$
2	0.62	88.3	7.6
4	1.2	93.8	15.2
6	1.8	95.8	22.7
8	2.5	96.8	30.3
11	3.4	97.7	41.7
14	4.3	98.2	53.1
$Vmid =$ 15.5	4.8	98.3	58.8
17	5.2	98.5	64.4
20	6.2	98.7	75.8
23	7.1	98.9	87.2
26	8.0	99	98.6
60	18	99.6	227.4
160	49	99.8	606.5

Table S1 Amounts of F4TCNQ titrants in terms of microliters and number of moles added to the photodoped ITO NCs solution. Molar ratio percentage is defined as mol % = 100 × moles of F4TCNQ / (moles of F4TCNQ + moles of ITO NCs). $n_{reacted}$ is the number of F4TCNQ molecules that reacted with each ITO NC.

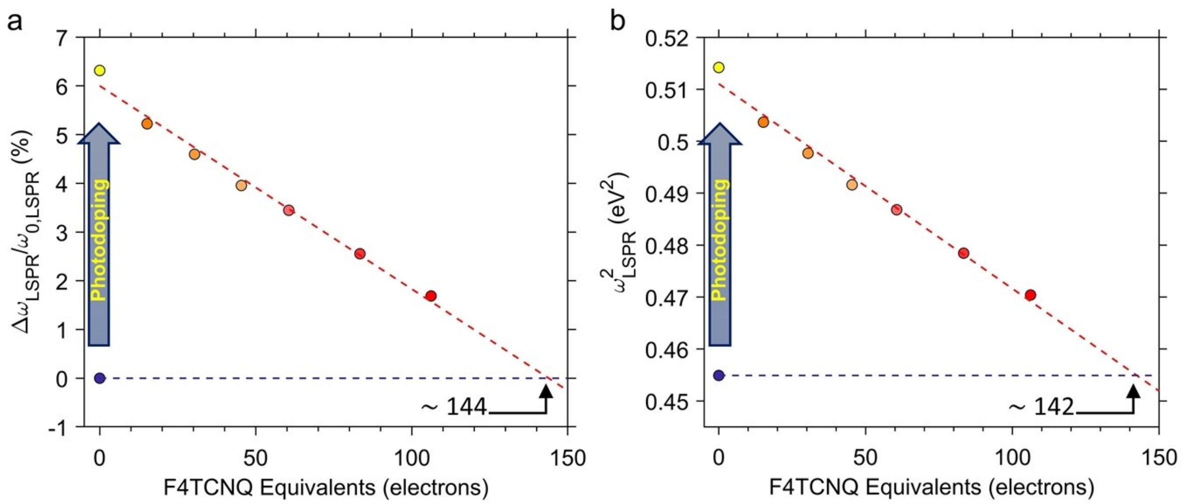

Figure S4 (a) Relative variation of the plasmon peak energy after photodoping and (b) plasmon peak energy squared as a function of F4TCNQ equivalents (range between 0 and $14\mu L$) In panel (a) $\Delta\omega_{LSPR}$ is defined as $\omega_{LSPR,titration} - \omega_{0,LSPR}$ being $\omega_{LSPR,titration}$ and $\omega_{0,LSPR}$ the plasmon peak energy after a titration step and before photodoping, respectively. F4TCNQ equivalents are obtained as the ratio between the number of moles of F4TCNQ and the number of moles of ITO NCs; this ratio is then multiplied by a factor 2 to account for the double electron transfer.
